# Circulation of influenza virus from 2009 to 2018 in Cameroon: 10 years of surveillance data

**DOI:** 10.1371/journal.pone.0225793

**Published:** 2019-12-03

**Authors:** Richard Njouom, Chavely Gwladys Monamele, Hermann Landry Munshili Njifon, Sebastien Kenmoe, Mohamadou Ripa Njankouo, the Network of influenza surveillance in Cameroon

**Affiliations:** Virology department, Centre Pasteur of Cameroon, Yaoundé, Cameroon; Icahn School of Medicine at Mount Sinai, UNITED STATES

## Abstract

Since the recent emergence of several subtypes of influenza viruses with pandemic potentials, there has been growing interest on the control of this infection worldwide. This study aimed to describe the 10 years of influenza activity in Cameroon between January 2009 and December 2018. Respiratory samples were collected from sentinel sites responsible for influenza surveillance in Cameroon and analyzed for the presence of influenza. Globally, 9 of the 10 administrative regions of the country were represented with at least 1 year of data. A total of 11816 respiratory samples were collected and influenza virus detection rate was 24.0%. The most represented age group was the 0–1 years representing more than 40% of the collected samples and possessing the lowest proportion of influenza cases (16.2%). Meanwhile higher proportions of influenza positive cases was found in the 2–4, 5–14 and 15–49 years age group at ≥29%. Among outpatients, the frequency of influenza virus was 24.8% while in hospitalized patients, 18.7% of samples were positive for influenza virus. We noted year-round circulation of influenza virus in Cameroon with 2 peaks in activity: a major peak in the months of September to December and a minor peak in the months of March to July. Antigenic characterization of influenza isolates showed 37.5% (6/16) vaccine match between the predominant Cameroon strains and the Northern hemisphere vaccine strains with majority of vaccine match observed in influenza B/Victoria subtype (4/6; 66.7%). Data collected from this surveillance system is essential to add to global information on the spread of influenza.

## Introduction

Influenza is a viral infection responsible for a significant cause of morbidity and mortality worldwide. Globally, the World Health Organization (WHO) estimates that annually severe cases due to influenza are estimated at 3 million with 290.000 to 650.000 deaths [1]. Influenza activity varies with respect to the geographical zone. In temperate regions, influenza circulates throughout the year with a marked increase in cases recorded during winter periods [1]. However, in tropical and subtropical regions influenza activity is noted year-round with peaks during the rainy season [2–4]. Since the recent emergence of several subtypes of influenza viruses with pandemic potentials, there has been growing interest on the control of this infection worldwide.

As a control measure, influenza surveillance has been established in several countries for monitoring circulating strains, detecting emerging viruses, studying epidemiological trends and to define seasonality in different geographical areas. Also, influenza surveillance is relevant for the evaluation of antigenic and genetic characteristics of circulating strains in comparison with recommended vaccine strains [4].

In Cameroon, influenza surveillance started in 2007 and results of the first year of activity have been reported with a positivity rate of 29% between November 2007 and October 2008 [5]. Since then, there has been no report on influenza activity within the surveillance system. This study aimed to describe 10 years of influenza surveillance data in Cameroon between January 2009 and December 2018 in order to obtain current information on the circulation of influenza, to better understand the epidemiology of the virus and to compare Cameroon isolates to previously recommended vaccine strains.

## Materials and methods

### Description of the influenza surveillance system in Cameroon

**Fig 1:**
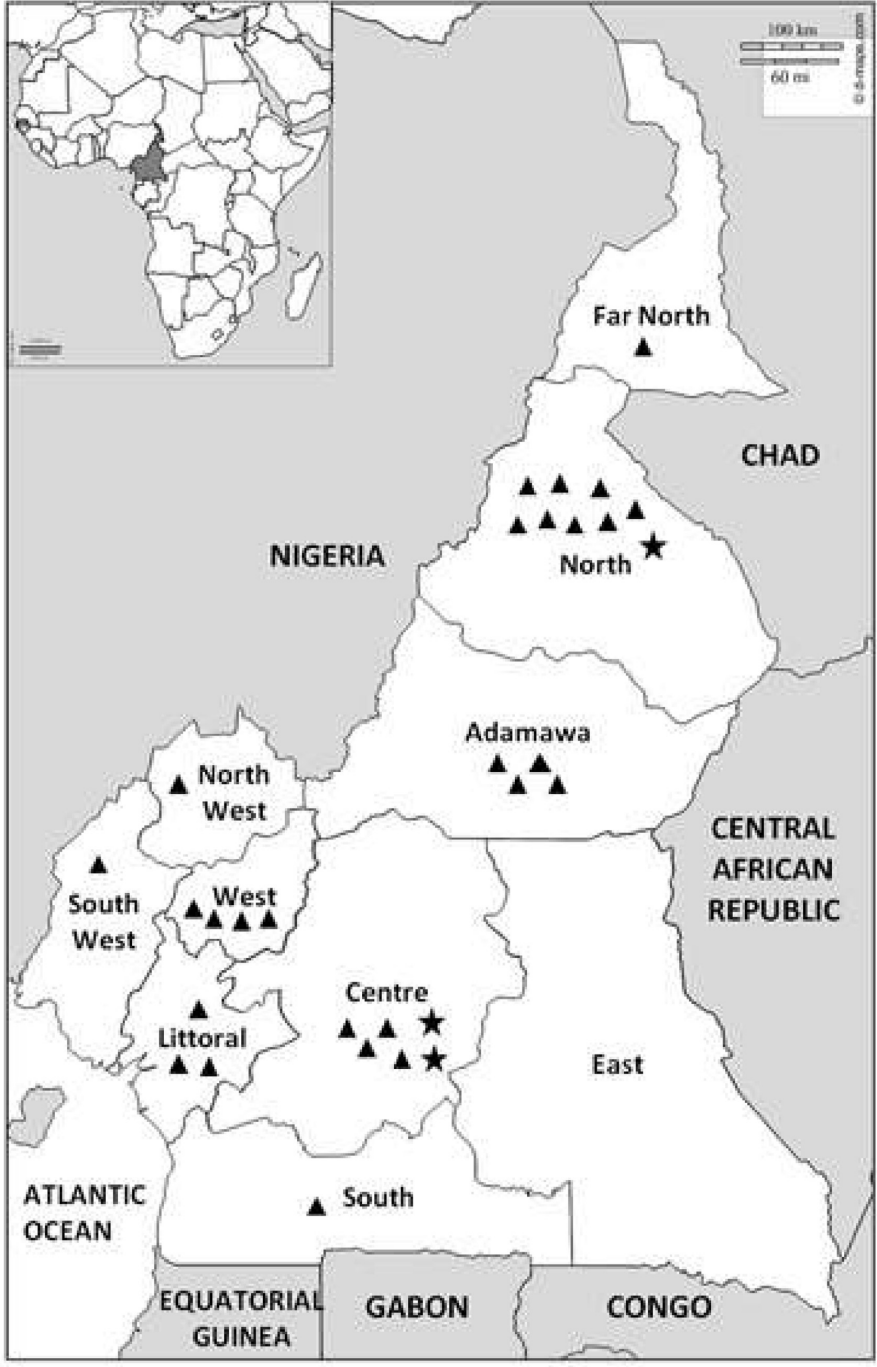
Presentation of sentinel sites by region. Triangles represent ILI sites while stars represent SARI sites.

The Centre Pasteur of Cameroon (CPC) has implemented an influenza surveillance system since November 2007initially involving sentinel sites in the Centre region of the country. In January 2009, the surveillance system extended to three other regions and CPC was designated the National Influenza Centre (NIC) by the Cameroon Ministry of Health and by the World Health Organisation. Sentinel sites were progressively added or reduced in the system based on their engagement and with respect to the quality of data reported. The number of sites represented in each year varied from as low as 10 in 2011 to as high as 22 in 2015–2016 (Table 1; Fig 1). Globally, 9 of the 10 administrative regions of the country were represented with at least 1 site and a minimum of 1 year data. Criteria for selection of the sentinel sites included willingness of the administrative and clinical staff of the hospital to participate, ease of sample transportation to the reference laboratory, size of catchment area of the hospital, and geographical location of the sites. Two main types of sites can be identified in the surveillance system of Cameroon; ILI (influenza-like illness) sites collect data from outpatients while SARI (severe acute respiratory infection) sites collect data exclusively from hospitalized patients.

**Table 1:** Temporal and regional distribution of sentinel sites in Cameroon.

Region	Site name	Distribution over years									
		2009 (14)	2010 (11)	2011 (10)	2012 (11)	2013 (11)	2014 (20)	2015 (22)	2016 (22)	2017 (16)	2018 (16)
Centre (6)	CMA de Nkomo	+	+	+	+	+	+	+	+		
	CASS de Nkolndongo	+	+	+	+	+	+	+	+	+	+
	CMS Ambassade de France	+	+	+	+	+	+	+	+	+	+
	CSI d’Etoudi	+	+	+	+	+	+	+	+	+	+
	Centre Hospitalier d’Essos*	+	+	+	+	+	+	+	+	+	+
	Hôpital Jamot de Yaoundé*									+	+
North (9)	Hôpital Provincial de Garoua	+									
	CSI de Koléré	+	+	+	+	+	+				
	CSI de Ourokanadi						+	+	+		
	CSI de Poumpouré						+	+	+		
	CSI de Souari						+	+	+		
	CSI de Kotta Liddiri						+	+	+	+	+
	Hôpital de Foulbéré						+	+	+	+	+
	CSI de Roumde Adjia						+	+	+	+	+
	Garoua Regional Hospital*									+	+
West (4)	CMA Koukouet Maloum	+									
	CMA Djeleng	+	+								
	CSI de Bandjoun	+	+	+	+	+	+	+	+	+	+
	CSI de Kueka (Foumban)	+	+	+	+	+	+	+	+	+	+
Littoral (4)	Hôpital Catholique Barcelone	+									
	Hôpital de District New Bell Pédiatrie	+	+	+							
	Hôpital d’Albert le Grand	+	+	+	+	+	+	+	+	+	+
	Hôpital Catholique de Log Pom				+	+	+	+	+	+	+
Far North (1)	Hôpital de Kolofata				+	+					
Adamawa (4)	CSI de Boumdjéré						+	+	+		
	CSI de Marza						+	+	+		
	CSI de Sabongari						+	+	+		
	Hopital Protestant de Ngaoundéré						+	+	+		
South (1)	Ebolowa Regional Hospital							+	+	+	+
South West (1)	Mount Mary Hospital Buea							+	+	+	+
North West (1)	Polyclinic St Blaise Bamenda							+	+	+	+

*SARI sites; In brackets () are the number of sites represented in each region or year

### Data collection at sentinel sites

Respiratory samples (nasopharyngeal and/or oropharyngeal swabs) were collected in 2mL cryovials containing viral transport medium from patients with clinical evidence of ILI, or from patients with SARI in all sentinel sites involved in influenza surveillance. ILI and SARI cases were identified based on the WHO definition. ILI was defined by patients presenting fever with cough and/ or sore throat within 5 days (10 days since 2016) while SARI had an additional requirement of necessitating hospitalisation as compared to the ILI case definition. Samples was collected from all individuals respecting the case definition and stored in the refrigerator at 4°C. The samples were then transported once a week to the NIC at CPC where all analyses were performed. Transport of samples from sentinel sites was performed with a cooler and through travel agencies for sites located out of the Centre region. This study was conducted as part of the influenza surveillance activity which is coordinated by Centre Pasteur of Cameroon, the National Influenza Centre. Individual ethical approval was therefore not required at inclusion. However, sample collection proceeded only after acceptance by the person or guardian and after written informed consent was provided. All individual data were anonymized with a code and were entered in a password protected database in Microsoft Office Access.

### Extraction of viral RNA and detection of influenza virus

The QIAamp Viral RNA Mini Kit (Qiagen, Hilden, Germany) was used to extract RNA from clinical samples into a final volume of 60 μL elution buffer following the manufacturer’s instructions. Extracts were analyzed for the presence of influenza using a Real-time RT-PCR assay, the CDC Influenza A/B Typing Panel RUO (CDC, Atlanta, GA, USA) in an ABI Prism 7300 or 7500 thermocycler (Applied Biosystems, Foster City, California, USA). Positive samples were subsequently sub-typed using the CDC Influenza A (H3/H1 pdm09) Subtyping Panel RUO (CDC, Atlanta, GA, USA). All PCR reactions were performed with the following enzymes: Invitrogen SuperScript^™^ III Platinum One-step Quantitative RT-PCR System (ThermoFisher Scientific, Massachusetts, USA) or Ambion AgPath-ID^™^ One-Step RT-PCR Kit (ThermoFisher Scientific, Massachusetts, USA). Samples were considered positive for influenza when threshold cycles (Ct) below 37 were observed.

### Haemagglutination inhibition assay

A representative number of samples that tested positive for influenza by real-time RT-PCR methods were sent each year to the WHO Collaborating Center (WHOCC) for Reference and Research on Influenza in London where antigenic characterization by Haemagglutination Inhibition assay was performed. In brief, 200 μL of each specimen was inoculated onto Madin-Darby canine kidney (MDCK) cells, with 2 ml per well of maintenance medium containing TPCK trypsin at a concentration of 2.0 mg/ml, in a 6-well plate. The plates were incubated at 37°C in a 5% CO_2_ atmosphere for 1 week to assess cytopathic effects. The influenza isolates were characterized by a hemagglutination inhibition assay using reference viruses (Table 2). Antigenic characterization of each subtype was determined based on the HI titers observed with respect to the reference viruses. Mismatch was defined here as the difference observed between the predominant antigenic characterization for a particular subtype and the Northern hemisphere vaccine strain for a given year.

**Table 2:** Reference viruses for haemagglutination inhibition assay.

Virus Type	Reference strains	Year
A(H3N2)	A/Wisconsin/67/2005	2009
	A/Brisbane/10/2007	2009
	A/Uruguay/716/2007	2009
	A/Finland/9/2008	2009
	A/Johannesburg/15/2008	2009
	A/Hong Kong/1952/2009	2009
	A/Hong Kong/1985/2009	2009
	A/Perth/16/2009	2013–2014
	A/Stockholm/18/2011	2013
	A/Iowa/19/2010	2013
	A/Victoria/361/2011	2013–2014
	A/Athens/112/2012	2013
	A/Texas/50/2012	2013–2015
	A/Samara/73/2013	2013–2015
	A/Serbia/NS210/2013	2013
	A/Hong Kong/146/2013	2013–2015
	NIB-85 (A/Almaty/2958/2013)	2013
	A/South Africa/4655/2013	2014
	A/Stockholm/6/2014	2014–2015
	A/Norway/466/2014	2014
	A/Netherlands/525/2014	2015
	A/Hong Kong/5738/2014	2015
	A/Hong Kong/4801/2014	2015
	A/Switzerland/9715293/2013	2014–2015
	A/Georgia/532/2015	2015
A(H1N1)pdm09	A/California/4/2009	2010
	A/California/7/2009	2010, 2011, 2013, 2015, 2018
	A/England/195/2009	2010
	A/Auckland/3/2009	2010
	A/Bayern/69/2009	2010, 2011, 2013, 2015, 2018
	A/Lviv/N6/2009	2010, 2011, 2013, 2015, 2018
	A/Hong Kong/2212/2010	2010
	A/Christchurch/16/2010	2010, 2011, 2013, 2015
	A/Hong Kong/3934/2011	2011, 2013
	A/Astrakhan/1/2011	2011, 2013, 2015, 2018
	A/St. Petersburg/27/2011	2011, 2013, 2015, 2018
	A/St. Petersburg/100/2011	2011, 2013, 2015
	A/Hong Kong/5659/2012	2013, 2015, 2018
	A/South Africa/3626/2013	2013, 2015, 2018
	A/Slovenia/2903/2015	2015, 2018
	A/Michigan/45/2015	2017
	A/Israel/Q-504/2015	2018
	A/Paris/1447/2017	2018
B/Victoria	B/Shandong/7/97	2010
	B/Malaysia/2506/2004	2010–2011, 2014, 2016
	B/Victoria/304/2006	2010
	B/England/393/2008	2010–2011
	B/Brisbane/60/2008	2010–2011, 2014, 2016
	B/Paris/1762/2008	2010–2011, 2014
	B/Hong Kong/514/2009	2010–2011, 2014,2016
	B/Odessa/3886/2010	2010–2011, 2014
	B/Malta/636714/2011	2014, 2016
	B/Johannesburg/3964/2012	2014, 2016
	B/Formosa/V2367/2012	2014, 2016
	B/South Australia/81/2012	2014, 2016
	B/Ireland/3154/2016	2016
	B/Nordrhein-Westfalen/1/2016	2016
B/Yamagata	B/Florida/4/2006	2013, 2016
	B/Brisbane/3/2007	2013, 2016
	B/Wisconsin/1/2010	2013, 2016, 2018
	B/Stockholm/12/2011	2013, 2016, 2018
	B/Estonia/55669/2011	2013, 2016, 2018
	B/Novosibirsk/1/2012	2013
	B/Hong Kong/3577/2012	2013
	B/Massachusetts/02/2012	2013, 2016, 2018
	B/Phuket/3073/2013	2016, 2018
	B/Hong Kong/3417/2014	2016
	B/Mauritius/1791/2017	2018

### Statistical analysis

Association between influenza positivity rate and some socio-demographic data was determined using binary logistic regression in R version 3.5.2. A reference population was considered for each category of interest and was used for comparisons in order to calculate the odds of infection. P values below 0.05 were considered statistically significant.

## Results

### Study population and proportion of influenza cases

The overall influenza virus detection rate during the 10 years of surveillance activity was 24.0% (Table 3); with 38.0% accounting for A(H3N2), 33.2% for influenza B, 20.8% for A(H1N1)pdm09, 6.4% for untyped A, 1.3% for A/B co-infection and 0.1% for A(H1N1). Influenza virus positivity rate varied from as low as 17.8% in 2014 to as high as 31.2% in 2018. Predominance of influenza sub-types varied with respect to the year of collection: A(H3N2) was the predominant sub-type in 2009, 2012, 2015 and 2018; A(H1N1)pdm09 was predominant in 2010, 2013 and 2017; while influenza B was predominant in the years 2011, 2014 and 2016.

**Table 3:** Proportion of influenza positive cases between 2009 and 2018.

	No. tested	Influenza virus positive N (%)	OR	P-value	Distribution of influenza virus type/ subtype					
					A(H3N2) N (%)	A(H1N1)pdm09 N (%)	A(H1N1) N (%)	A untyped N (%)	B N (%)	A and B N (%)
**Age group / years**										
0–1	4884	791 (16.2)	Ref	Ref	288 (36.4)	157 (19.8)	0	53 (6.7)	281 (35.5)	12 (1.5)
2–4	2823	818 (29.0)	2.11	<0.001	301 (36.8)	196 (24.0)	1 (0.1)	62 (7.6)	251 (30.7)	7 (0.9)
5–14	1464	467 (31.9)	2.42	<0.001	155 (33.2)	84 (18.0)	2 (0.4)	22 (4.7)	200 (42.8)	4 (0.9)
15–49	1443	431 (29.9)	2.20	<0.001	190 (44.1)	85 (19.7)	1 (0.2)	20 (4.6)	131 (30.4)	4 (0.9)
50–64	202	53 (26.2)	1.84	<0.001	29 (54.7)	6 (11.3)	0	3 (5.7)	14 (26.4)	1 (1.9)
≥ 65	101	27 (26.7)	1.88	0.005	16 (59.3)	1 (3.7)	0	1 (3.7)	7 (25.9)	2 (7.4)
No data	899	248 (27.6)			100 (40.3)	61 (24.6)	0	20 (8.1)	59 (23.8)	8 (3.2)
**Gender**										
Male	5459	1261 (23.1)	0.94	0.191	474 (37.6)	261 (20.7)	3 (0.2)	78 (6.2)	427 (33.9)	18 (1.4)
Female	5392	1303 (24.2)	Ref	Ref	492 (37.8)	266 (20.4)	1 (0.1)	82 (6.3)	450 (34.5)	12 (0.9)
No data	965	271 (28.1)			113 (41.7)	63 (23.2)	0	21 (7.7)	66 (24.4)	8 (3.0)
**Type of illness**										
ILI	10193	2531 (24.8)	1.43	<0.001	960 (37.9)	501 (19.8)	3 (0.1)	153 (6.0)	878 (34.7)	36 (1.4)
SARI	1547	290 (18.7)	Ref	Ref	110 (37.9)	86 (29.7)	0	27 (9.3)	65 (22.4)	2 (0.7)
No data	76	14 (18.4)			9 (64.3)	3 (21.4)	1 (7.1)	1 (7.1)	0	0
**Duration of illness**										
0–5	9230	2343 (25.4)	Ref	Ref	912 (38.9)	489 (20.9)	3 (0.1)	143 (6.1)	766 (32.7)	30 (1.3)
6–10	1186	234 (19.7)	0.72	<0.001	74 (31.6)	55 (23.5)	0	12 (5.1)	86 (36.8)	7 (3.0)
>10	465	70 (15.1)	0.52	<0.001	26 (37.1)	12 (17.1)	0	7 (10.0)	24 (34.3)	1 (1.4)
No data	935	188 (20.1)			67 (35.6)	34 (18.1)	1 (0.5)	19 (10.1)	67 (35.6)	0
**Region**										
Centre	6474	1604 (24.8)	Ref	Ref	678 (42.3)	305 (19.0)	2 (0.1)	106 (6.6)	493 (30.7)	20 (1.2)
Adamawa	459	70 (15.3)	0.55	<0.001	25 (35.7)	25 (35.7)	0	3 (4.3)	17 (24.3)	0
Far North	60	13 (21.7)	0.84	0.579	11 (84.6)	2 (15.4)	0	0	0	0
Littoral	1173	359 (30.6)	1.34	<0.001	128 (35.7)	66 (18.4)	0	19 (5.3)	137 (38.2)	9 (2.5)
North	1566	386 (24.6)	0.99	0.917	68 (17.6)	111 (28.8)	0	28 (7.3)	176 (45.6)	3 (0.8)
North West	332	73 (22.0)	0.86	0.251	24 (32.9)	11 (15.1)	0	12 (16.4)	25 (34.2)	1 (1.4)
South	216	60 (27.8)	1.17	0.316	19 (31.7)	23 (38.3)	0	2 (3.3)	15 (25.0)	1 (1.7)
South West	316	70 (22.2)	0.86	0.291	37 (52.9)	11 (15.7)	0	2 (2.9)	17 (24.3)	3 (4.3)
West	1220	200 (16.4)	0.60	<0.001	89 (44.5)	36 (18.0)	2 (1.0)	9 (4.5)	63 (31.5)	1 (0.5)
**Total**	**11816**	**2835 (24.0)**			**1079 (38.0)**	**590 (20.8)**	**4 (0.1)**	**181 (6.4)**	**943 (33.3)**	**38 (1.3)**

Association between influenza positivity rate and some socio-demographic data was determined, that is: age group, type of illness, period of illness and region. All socio-demographic factors were significantly associated to positive influenza cases with P-values below 0.05 except gender. Both genders were equally represented with a slightly higher influenza positivity rate in females (24.2%) than males (23.1%) and A(H3N2) was the predominant sub-type detected in both populations.

The most represented age group was aged 0–1 years; it represented 41.3% (4884/11816) of the collected samples and possessed the lowest proportion of influenza cases (16.2%). While higher proportions of influenza positive cases were found in the 2–4, 5–14 and 15–49 years age group at ≥29%. In all age groups influenza A(H3N2) was the most frequently detected type at 36.4–59.3% except in the 5–14 years age group where influenza B was the predominant type (42.8%). In ILI cases, the frequency of influenza virus was 24.8% while in SARI cases 18.7% of the samples were positive for influenza virus. The proportion of SARI in each age group in ascending order was respectively: 15.1% (736/4884), 13.3% (376/2805), 12.1% (177/1460), 7.6% (110/1435), 12.9% (26/200) and 28.7% (29/101). The elderly population had the highest proportion of SARI cases. Majority of positive influenza cases had their samples collected within 5 days of onset of illness while the lowest proportion of influenza cases were noted in specimens collected after 10 days following disease onset.

Regarding the regions, the Centre region had the highest proportion of collected samples (54.8%; 6474/11816) with an influenza positivity rate of 24.8%. Similarly, the least number of samples were obtained from the Far North region (0.5%; 60/11816) with 21.7% of influenza cases. In comparison to the Centre region, influenza virus detection rate was higher in the Littoral region and lower in the Adamawa and West regions. In most regions, the dominant influenza subtype was A(H3N2); however, influenza B was predominant in the Littoral, North and North West regions while A(H1N1)pdm09 was predominant in the South region.

### Trends in influenza positivity rate

**Fig 2:**
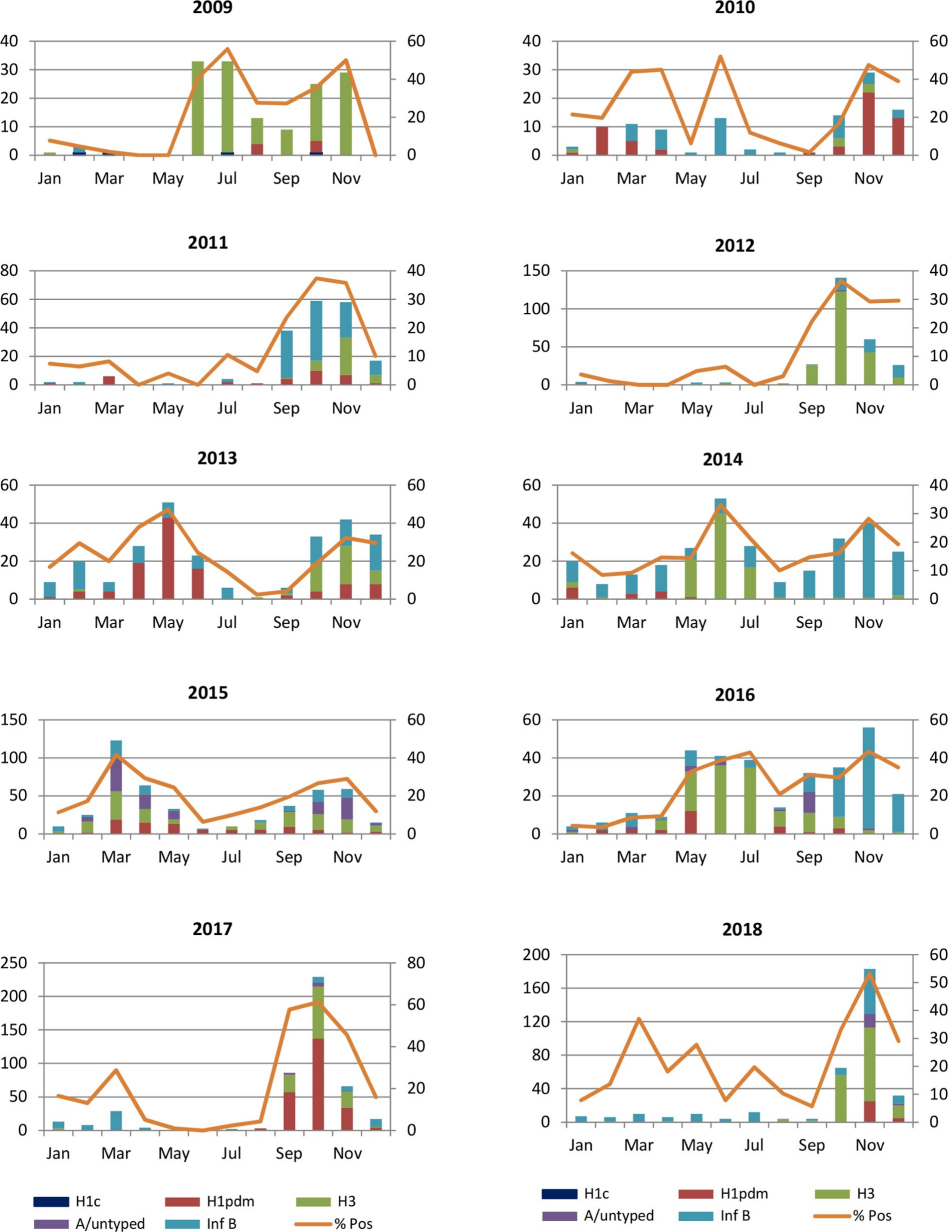
Distribution and trends in influenza virus positivity rate from 2009–2018. The primary y-axis represents the frequency of each subtype; the secondary y-axis represents the percentage positive for influenza and the x-axis represents the months.

We noted year-round circulation of influenza virus in Cameroon with increased activity depending on distinct periods of the year. Following the epidemic threshold which was set at 20% in Cameroon, two peaks were observed in influenza activity: a major peak was noted by the end of the year in the months of September to December and a minor peak was noted in the months of March to July (Fig 2). Majority of the years were characterized by peaks in influenza A(H3N2) and influenza B in the months of September to December. However, in 2010 there was year round circulation of influenza A(H1N1)pdm09 and influenza B. Meanwhile, 2017 was characterized by the predominance of influenza A(H1N1)pdm09 and A(H3N2) from September to November.

**Fig 3:**
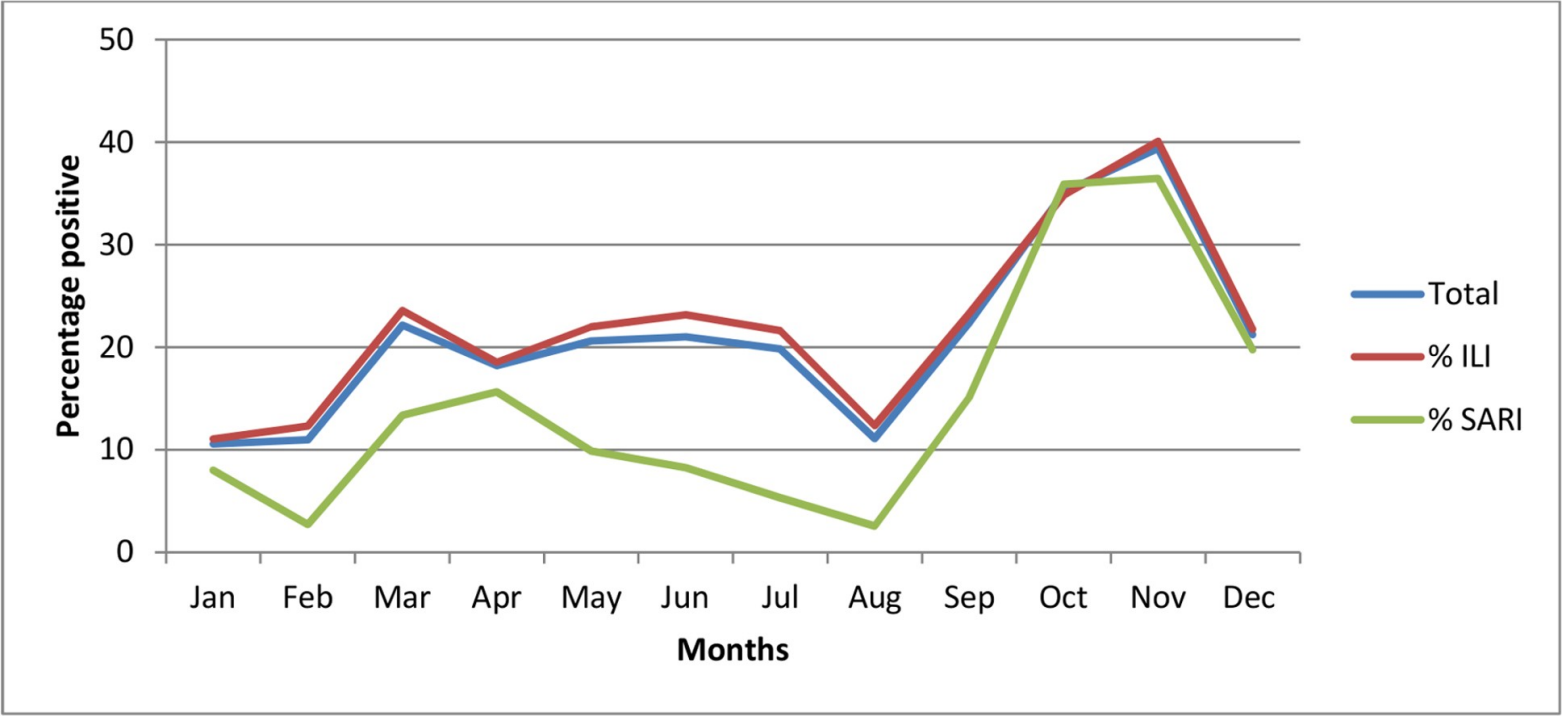
Influenza virus positivity rate among ILI and SARI cases. Total represents the overall percentage positive for influenza; %ILI represents the percentage positive for influenza among patients with ILI; %SARI represents the percentage positive for influenza among patients with SARI.

The trend in influenza positivity rate among SARI cases was similar to that observed in ILI cases, although, lower proportions were observed in the latter (Fig 3).

### Antigenic characterization of Cameroon strains and comparison with vaccine strains

Antigenic characterization of influenza isolates from Cameroon was performed and haemagglutination inhibition (HI) assay was carried out using reference anti-sera. Table 3 summarizes the results obtained from 2009–2018. Results of HI assay for subtypes below 5 counts were not considered due to multiple match with different reference anti-sera. Data was obtained for 1 to 3 influenza types per year summing up to 16 available data. Of these, we noted 6/16 vaccine match between the predominant Cameroon strains and the Northern hemisphere vaccine strain (Table 4). Majority of vaccine mismatch was noted for influenza A viruses (A(H1N1)pdm09 and A(H3N2)). With respect to influenza B viruses, B/Victoria was the predominant virus which showed vaccine match with the Northern hemisphere vaccine strain (4/6). However, in 2014 and 2016, the victoria lineage was included only in the quadrivalent vaccine composition.

**Table 4:** Antigenic characterization of influenza strains in Cameroon and comparison to vaccine strains.

Year	Virus Type	Predominant Cameroon strains	n/N	%	Northern Hemisphere vaccine strain	Vacine Match*
2009	A(H3N2)	A/Uruguay/716/2007 (A/Brisbane/10/2007-like)	25/25	100	A/Brisbane/10/2007	Yes
2010	A(H1N1)pdm09	A/Auckland/3/2009 (A/California/7/2009-like)	73/90	81.1	/	/
	B/Victoria	B/Hong Kong/514/2009 (B/Brisbane/60/2008-like)	93/102	91.2	B/Brisbane/60/2008	Yes
2011	A(H1N1)pdm09	A/St. Petersburg/100/2011	20/22	90.9	A/California/7/2009	No
	B/Victoria	B/Paris/1762/2008 (B/Brisbane/60/2008-like)	25/26	96.2	B/Brisbane/60/2008	Yes
2013	A(H3N2)	A/Samara/73/2013	12/16	75	A/Victoria/361/2011	No
	A(H1N1)pdm09	A/St. Petersburg/100/2011	27/27	100	A/California/7/2009	No
	B/Yamagata	B/Hong Kong/3577/2012	31/33	93.9	B/Wisconsin/1/2010	No
2014	A(H3N2)	A/Stockholm/6/2014	11/11	100	A/Victoria/361/2011	No
	B/Victoria	B/Hong Kong/514/2009 (B/Brisbane/60/2008-like)	7/11	63.3	B/Brisbane/60/2008**	Yes
2015	A(H3N2)	A/Stockholm/6/2014	5/5	100	A/Texas/50/2012	No
	A(H1N1)pdm09	A/St. Petersburg/100/2011	21/21	100	A/California/7/2009	No
2016	B/Yamagata	B/Hong Kong/3417/2014	5/6	83.3	B/Phuket/3073/2013	No
	B/Victoria	B/Ireland/3154/2016 (B/Brisbane/60/2008-like)	24/25	96	B/Brisbane/60/2008**	Yes
2017	A(H1N1)pdm09	A/Slovenia/2903/2015	18/18	100	A/Michigan/45/2015	No
2018	B/Yamagata	B/Phuket/3073/2013	19/19	100	B/Phuket/3073/2013	Yes

**quadivalent vaccine strain

* match between predominant Cameroon strains and Northern hemisphere vaccin strain

(/) = not applicable

n/N represents the proportion of Cameroon strains showing highest titers for the reference strains

## Discussion

We describe here the largest report of influenza sentinel surveillance from Central Africa with results of 10 years of activity. The overall influenza detection rate was 24.0% with A(H3N2) accounting for the majority of cases. Influenza virus positivity rate varied from as low as 17.8% in 2014 to as high as 31.2% in 2018. A previous report by Njouom et al. noted influenza positivity rate of 29% from November 2007 to October2008 within the same surveillance system [6].

The most represented age group was the 0–1 years accounting for more than 40% of the collected samples and possessing the lowest proportion of influenza cases. The high representation of the young population is due to the fact that seeking for medical attention is higher in children irrespective of their clinical condition as compared to the older population. Meanwhile, higher proportions of influenza positive cases were found in the 2–4, 5–14 and 15–49 years age group. Higher detection rates in these groups can be attributed to high transmission in schools due to high contact rate with peers as reported elsewhere [2, 7–10] since majority represent the school going age. These results are contradictory to reports from Manirakiza et al. in Central Africa Republic where infants aged 0–6 months (8.8%) and people aged 15–50 years (11.0%) had higher proportions of influenza cases [11]. A similar report by Nzussouo et al. from West African countries reported that majority of ILI and SARI case-patients testing positive for influenza viruses were children aged 0–4 years [12]. In the latter, higher rates of influenza-associated illness in the 0–4 years age group can be attributed to a selection bias since more than 70% of samples collected were from children aged 0–14 years.

In mild cases of infection (ILI), the frequency of influenza cases was 24.8% as compared to 18.7% in severe cases (SARI). Higher proportions of positive influenza cases in ILI as compared to SARI have as well been reported by Mainassara et al. in Niger as well as several other West African countries [8, 12, 13]. We noted that lower influenza positivity rate in SARI patients could be attributed to delayed presentation in hospitals resulting in reduced viral loads and lower detection rates as suggested by Dalhatu et al. [8]. Indeed, higher proportions of specimens collected within 5 days of onset of illness were observed in ILI cases as compared to SARI.

As noted by Caini et al., influenza is characterized by a heterogenous pattern in tropical countries and the same pattern was observed in this study [14]. However, globally, two peaks were observed in influenza activity in Cameroon: a major peak was noted in the months of September to December and a minor peak was noted in the months of March to July. These two periods of increased activity correspond to the periods of rainy season in Cameroon suggesting that timely vaccination can be carried out in Cameroon during these periods especially in children of school-going age. These results are further supported by a recent study reporting the association between influenza activity and weather variables which showed that rainfall enabled forecast of influenza activity in Yaounde-Cameroon [15]. Past studies in tropical countries such as Ivory Coast, Thailand have as well reported that increased influenza activity is associated with periods of increased rainfall [16–18]. This can be explained by the fact that there is increased indoor crowding and human-human contact during rainfall leading to more contact or aerosol transmission of influenza virus [19].

Predominance of influenza types varied with respect to the year of collection and there was no period characterized by the circulation of specific influenza types, however, majority of the years were characterized by peaks in influenza A(H3N2) and B in the months of september to december. With regards to influenza A(H1N1)pdm09, the first case was reported in August 2009 and the following year, it was the predominant subtype in circulation (50.9%) showing a high transmission rate of influenza virus and the importance of restoring influenza surveillance systems in countries with the aim of rapidly detecting rise in activity or the detection of novel influenza types of public health importance.

Out of the data for which antigenic characterization was available, we noted 6/16 vaccine match between the predominant Cameroon strains and the Northern hemisphere vaccine strains. B/Victoria was the predominant virus which showed vaccine match with Northern hemisphere vaccine strain. However, in 2014 and 2016, the victoria lineage was included only in the quadrivalent vaccine and probably did not match the circulating strain since only trivalent vaccines are available in Cameroon. Seasonal influenza vaccination is generally recommended for population at risk of complication including children and elderly individuals [20]. While current levels of vaccine uptake are low, the apparently low degree of match during recent seasons between circulating viruses and those included in the Northern hemisphere vaccines suggest that currently licensed vaccines are not suitable for use in Cameroon. However, larger scale studies are required in order to confirm these results. The first limitation of this study was that respiratory samples collected initially were not representative of the country, because virological surveillance started in only one region before it was gradually extended to 9 regions. Also, over 60% of patients enrolled were situated in the Centre region. The high representation of the Centre region can be attributed to the fact that the NIC is located in the Centre region where there is more efficient follow-through of sentinel sites. Moreover, the Centre region had the highest number of sentinel sites. Also the presence of some missing data for some demographic variables might have biased the conclusions made Consequently, our findings might not be generalizable to the whole country. Of the 22 sentinel sites involved in the surveillance, 7 provided specimens throughout the 10-years surveillance period. This highlights the challenges of establishing sentinel sites for surveillance and demonstrates that commitment of the staff is an essential condition to consider when selecting sentinel sites. Furthermore, because of the lack of environmental data such as rainfall and temperature for each sentinel site, we were unable to assess changes in influenza positivity rate by region with varying climatological data.

## Conclusion

This study showed the year-round circulation of influenza virus in Cameroon with two peaks in activity. Moreover, we noted that all age groups were equally affected by influenza virus in the exception of the 0–1 years age group which showed lower odds of infection. The CPC has put in place a successful influenza virus surveillance system in Cameroon that has the ability to rapidly detect influenza viruses by real-time RT-PCR assays and currently covers majority of the administrative regions of the country. Data collected from this surveillance system adds to global information on the spread of influenza.
